# Microarray transcriptome datasets of maternal-zygotic *DNA methyltransferase 3aa*^−/−^ zebrafish during early developmental stages

**DOI:** 10.1016/j.dib.2023.108967

**Published:** 2023-02-09

**Authors:** Masaki Shirai, Nobuyoshi Shimoda, Haruko Takahashi, Kazuya Takayama, Yutaka Kikuchi

**Affiliations:** a Department of Biological Science, Graduate School of Science, Hiroshima University, Kagamiyama 1-3-1, Higashi-Hiroshima, Hiroshima 739-8526 Japan; b Laboratory of Molecular Analysis, Center for Core Facility Administration, National Center for Geriatrics and Gerontology, 7-430, Morioka, Obu, Aichi 474-8522, Japan; c Graduate School of Integrated Sciences for Life, Hiroshima University, Kagamiyama 1-3-1, Higashi-Hiroshima, Hiroshima 739-8526, Japan

**Keywords:** Microarray, Epigenetics, *Dnmt3aa*, Zebrafish, Development

## Abstract

DNA methylation is an epigenetic regulator mediated by DNA methyltransferases (Dnmts). The methylation is involved in control of gene expression in vertebrates. It has been reported that there are mainly two types of *de novo* Dnmts, Dnmt3a and Dnmt3b, in mammals. These two Dnmts function in DNA methylation in the distinct or overlapping genomic regions. The zebrafish homologs of mammalian Dnmt3a are Dnmt3aa and Dnmt3ab. We generated a maternal-zygotic *dnmt3aa* deficient mutant (MZ*dnmt3aa*) to identify the specific target regions for DNA methylation in the zebrafish genome and their function in the developmental process. Microarray analysis revealed alterations in gene expression by knock-out of *dnmt3aa* in early zebrafish development. Microarray datasets were produced from samples at five different developmental stages: 1–2 cell, shield, 5-somite, 1-day post fertilization (dpf), and 2 dpf. Herein, we present novel raw and processed transcriptome datasets generated by analysis of the MZ*dnmt3aa*^−/−^ mutant. The raw microarray data are available through the Gene Expression Omnibus (GEO), accession number GSE202646. These transcriptome data may be useful for comparing differences in gene expression among species of Dnmt3a mutants and for analyzing human diseases caused by DNMT3A such as acute myelogenous leukemia (AML).

**Specifications Table**

**Table:** 

Subject	Developmental Biology
Specific subject area	Transcriptome, Epigenetics, DNA methylation
Type of data	Five tables, seven figures
How the data were acquired	Cyanine-3 (Cy3) labeled cRNA was prepared and hybridized to *Danio rerio* (Zebrafish) Oligo Microarray V3 (Design ID: Agilent-026,437) (Agilent). After hybridization, slides were scanned on the Agilent SureScan Microarray Scanner (G2600D) using one color scan setting for array slides (Scan Area 61 × 21.6 mm, Scan resolution 3 μm, Dye channel was set to Green, and Green PMT was set to 100%.).
Data format	Raw and analyzed
Description of data collection	Total RNA was extracted from 20 pooled zebrafish eggs/embryos/larvae of five developmental stages. RNA Integrity Number (RIN) values for all RNA samples exceeded 9.0. Microarray datasets were obtained from three biological replicates.
Data source location	• *Institution:* Hiroshima University • *City/Town/Region:* Higashi-Hiroshima, Hiroshima • *Country:* Japan
Data accessibility	Repository name: Gene Expression Omnibus (GEO) Data identification number: GSE202646 for microarray Direct URL to data: https://www.ncbi.nlm.nih.gov/geo/query/acc.cgi?acc=GSE202646
Related research article	Not applicable

**Value of the Data**

• These transcriptome datasets provide the genes regulated by Dnmt3aa during early zebrafish development.
• Comparative dataset of gene expression alterations at five developmental stages.
• Since we have already reported that DNA methylation changes by *dnmt3aa* mutation at 2 dpf [1], the transcriptome data for the zebrafish MZ*dnmt3aa^−/−^* mutant are useful for detailed analysis of the relationship between DNA methylation and gene expression via Dnmt3aa.
• This transcriptome dataset is useful for examining the comparative gene expression of *Dnmt3a* mutants among vertebrates.
• This dataset may be useful for elucidating human diseases caused by DNMT3A.

## 1 Objective

DNA methylation is an epigenetic regulator mediated by DNA methyltransferases (Dnmts). This methylation is involved in the control of gene expression in vertebrates [2]. Two main types of *de novo* Dnmts, Dnmt3a and Dnmt3b, have been reported in mammals [3]. Zebrafish homologs of mammalian Dnmt3a are Dnmt3aa and Dnmt3ab [4]. The detailed expression patterns of *dnmt3aa* were investigated at different developmental stages. After fertilization, *dnmt3aa* was expressed as a maternal transcript [5]. After the maternal-to-zygotic transition, *dnmt3aa* is ubiquitously expressed, and its expression pattern changes from the whole embryo to specific tissues during development [6]. We have previously reported changes in DNA methylation using MZ*dnmt3aa*^−/−^ mutants [1]. However, changes in gene expression in the *dnmt3aa*-deficient state have not yet been investigated. Therefore, we performed microarray analysis of samples from five different developmental stages:1–2 cell, shield, 5-somite, 1 dpf, and 2 dpf. In zebrafish, hemangioblasts, both hematopoietic and endothelial precursor cells, are present at the somite stage [7]. Because our dataset includes data of timepoints after the somite stage, this zebrafish dataset could potentially be used to study human blood disease AML.

## 2 Data Description

Here, we present microarray data for wild-type (WT) and MZ*dnmt3aa*^−/−^ zebrafish. The MZ*dnmt3aa*^−/−^ zebrafish completely lacked the maternal function of Dnmt3aa, allowing the identification of genes that are regulated by methylation via Dnmt3aa during early development. To investigate the gene expression profile by knock-out of *dnmt3aa* at five different developmental stages, we performed a microarray analysis. The data are as follows:

Supplementary Table S1: Microarray data at 1–2 cell stage
Supplementary Table S2: Microarray data at the shield stage
Supplementary Table S3: Microarray data at the 5-somite stage
Supplementary Table S4: Microarray data at 1 dpf
Supplementary Table S5: Microarray data at 2 dpf

Microarray data have been deposited in the GEO under accession number GSE202646.

**Fig. 1:**
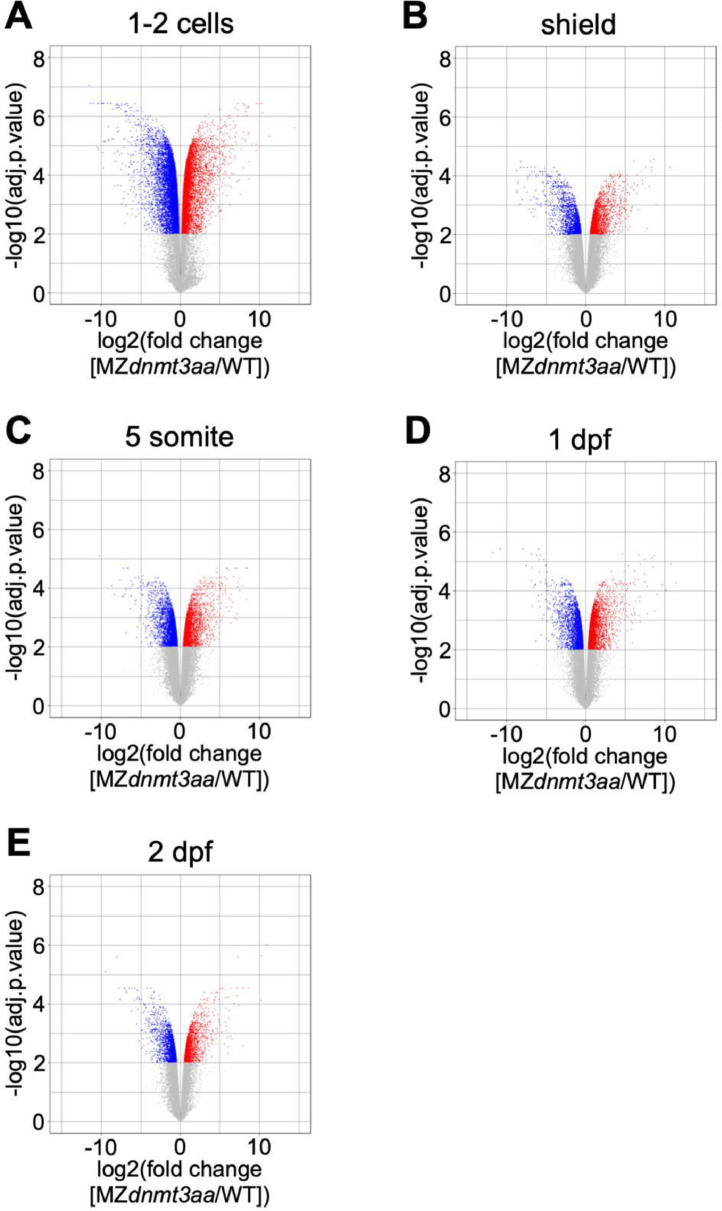
Volcano plots of differentially expressed genes based on samples of five developmental stages: (A) 1–2 cell, (B) shield, (C) 5-somite, (D) 1 dpf, and (E) 2 dpf. Red and blue dots indicate significantly up- and down-regulated genes, respectively. Gray dots are genes that have not changed significantly.

**Fig. 2:**
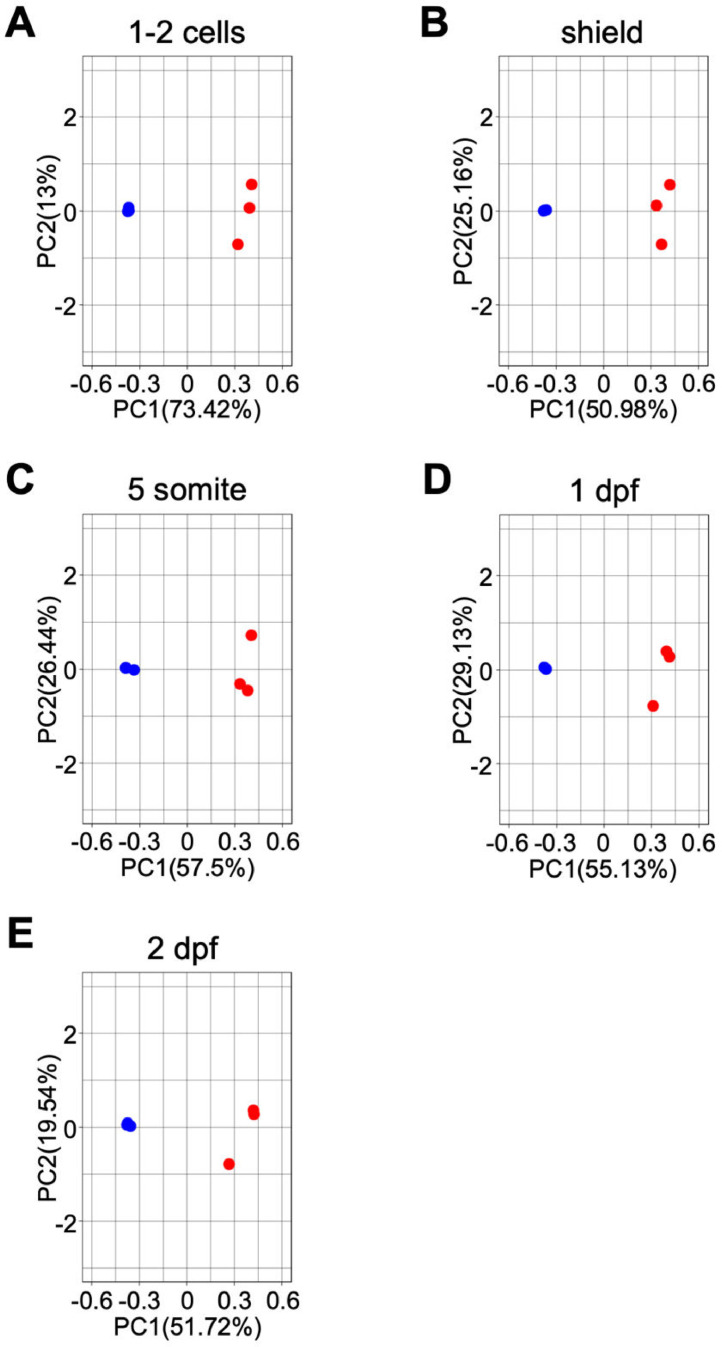
PCA of the data obtained from samples of 5 developmental stages: (A) 1–2 cell, (B) shield, (C) 5-somite, (D) 1 dpf, and (E) 2 dpf. Blue and red dots indicate WT and MZ*dnmt3aa*^−/−^ samples, respectively.

**Fig. 3:**
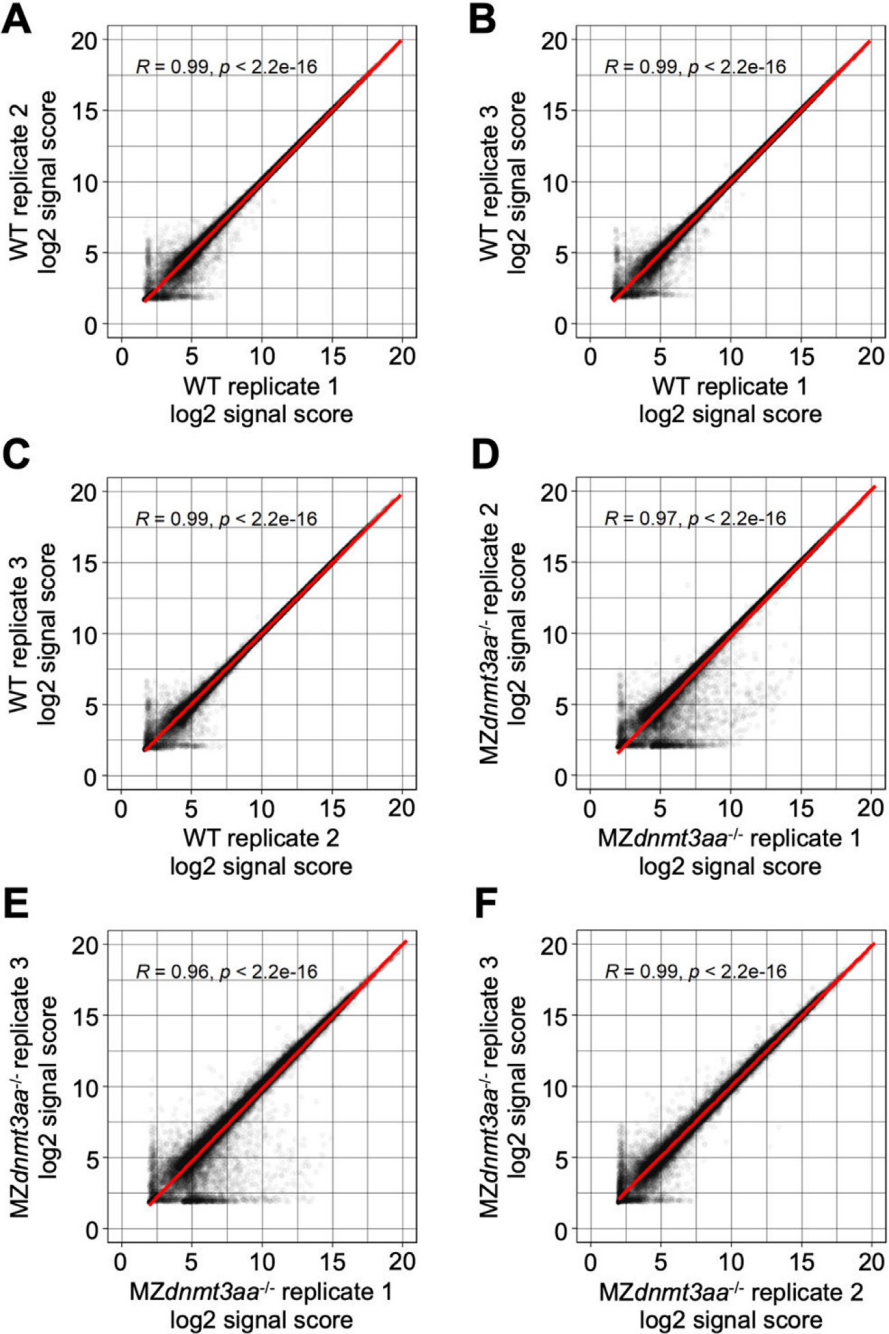
Correlation graph of log2 signal score between replications in 1–2 cell stage samples. Each dot indicates the signal score of each gene and the red line shows the linear regression.

**Fig. 4:**
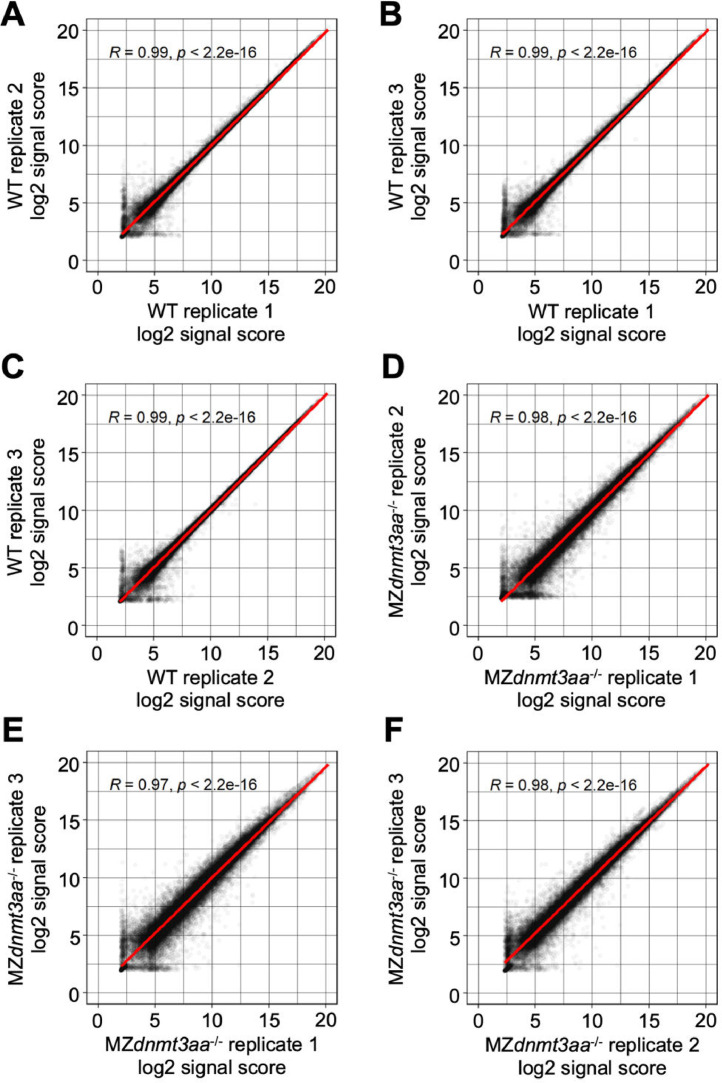
Correlation graph of log2 signal score between replications in shield stage samples. Each dot indicates the signal score of each gene and the red line shows the linear regression.

**Fig. 5:**
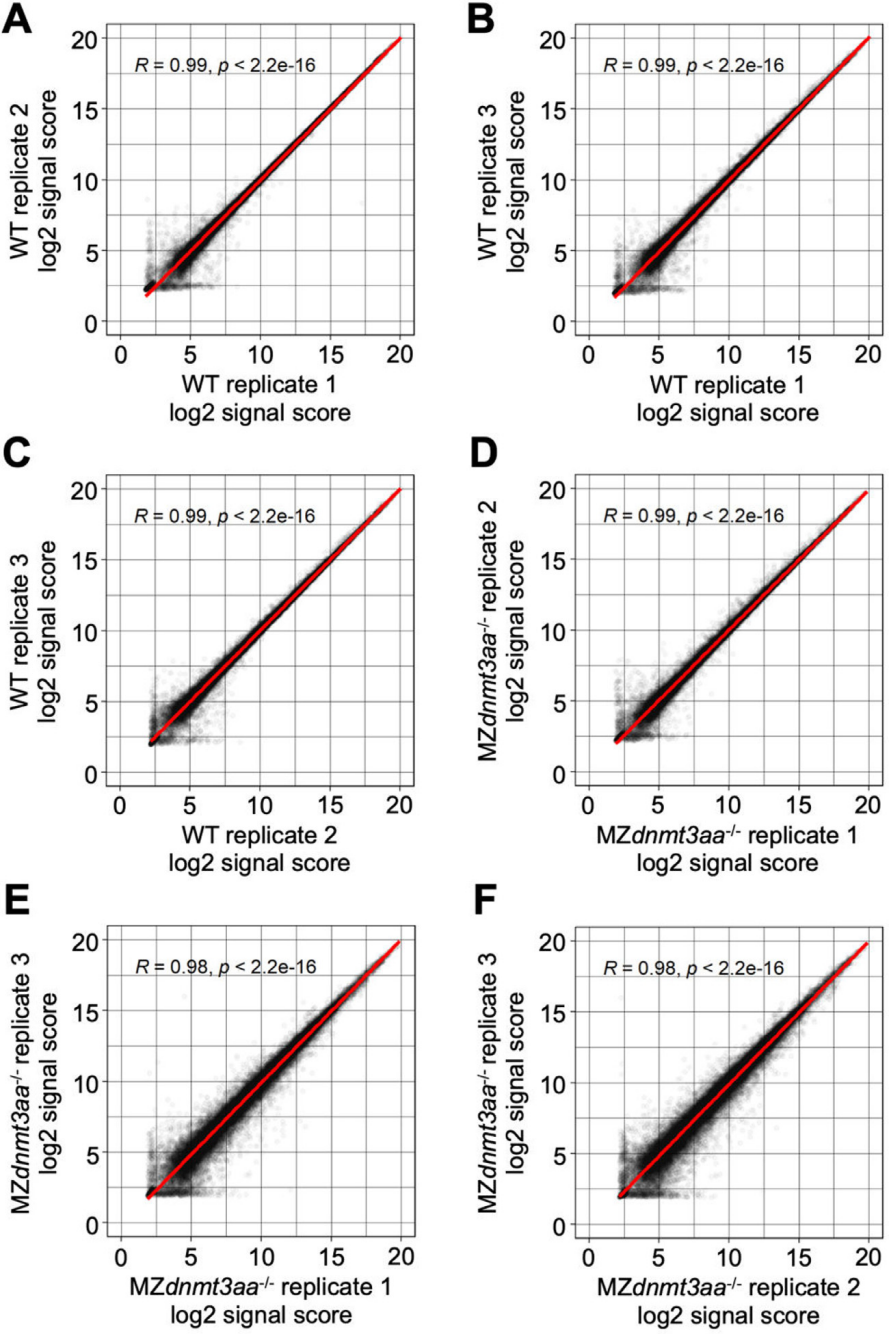
Correlation graph of log2 signal score between replications in 5-somite stage samples. Each dot indicates the signal score of each gene and the red line shows the linear regression.

**Fig. 6:**
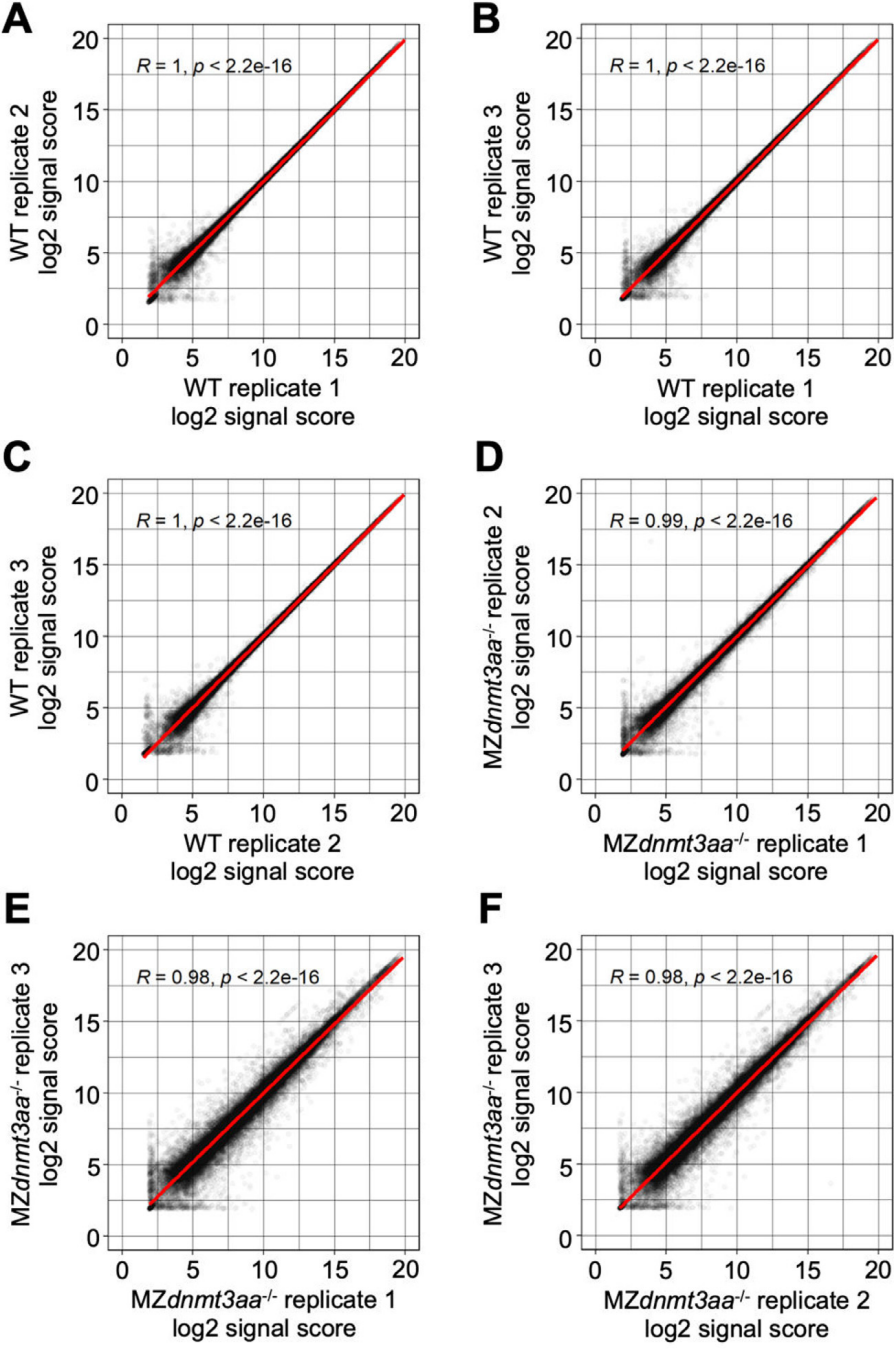
Correlation graph of log2 signal score between replications in 1 dpf stage samples. Each dot indicates the signal score of each gene and the red line shows the linear regression.

**Fig. 7:**
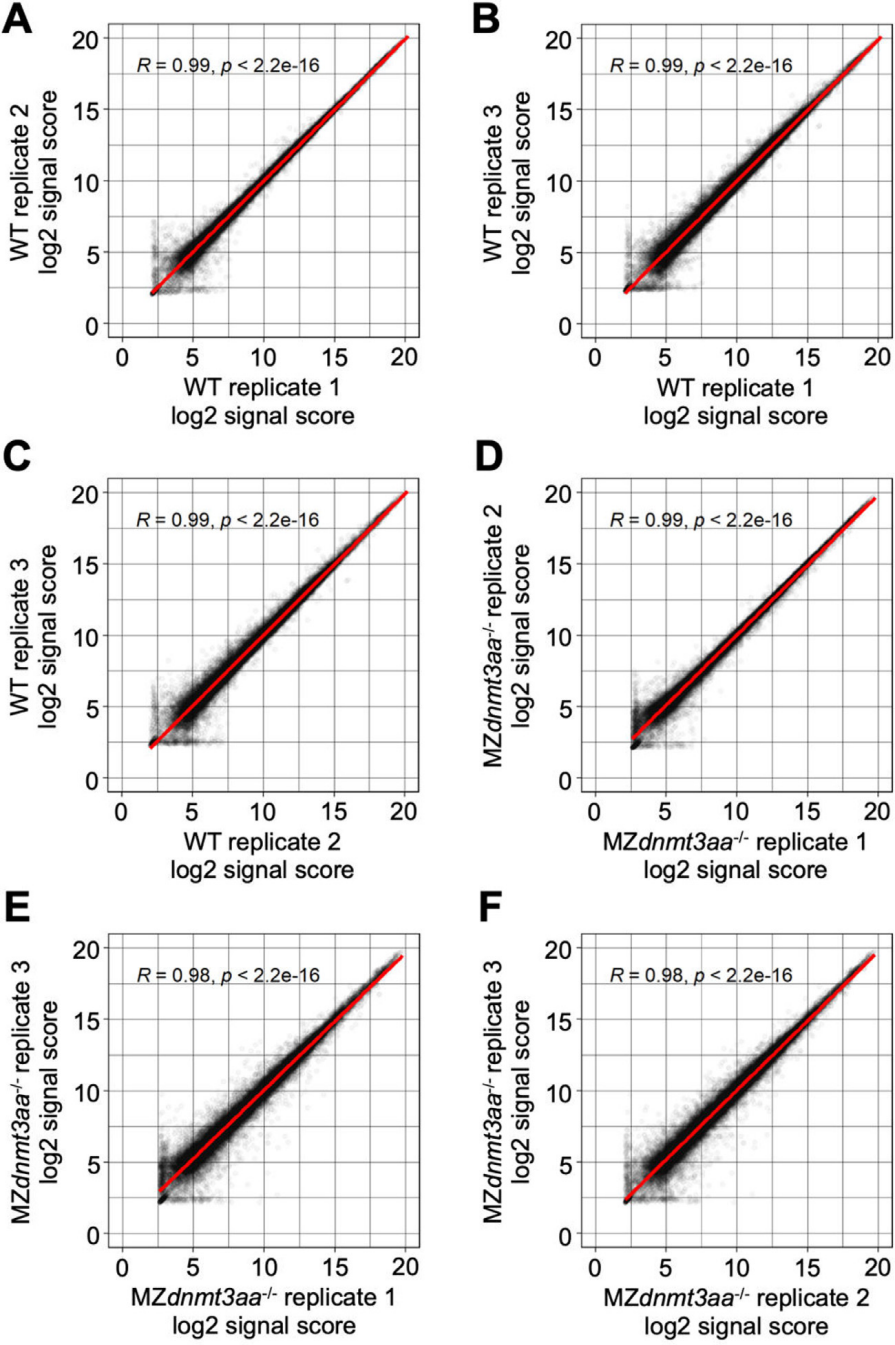
Correlation graph of log2 signal score between replications in 2 dpf stage samples. Each dot indicates the signal score of each gene and the red line shows the linear regression.

Volcano plots show the differentially expressed genes (DEGs) between WT and MZ*dnmt3aa*^−/−^ zebrafish (Fig. 1). Dataset quality was confirmed using principal component analysis (PCA) and Pearson correlation analysis for replicates of each developmental stage (Fig. 2–7).

## 3 Experimental Design, Materials and Methods

### 3.1 Microarray Sample Preparation and Hybridization

Zebrafish adults, larvae, and embryos were maintained and handled as previously described [8]. To extract total RNA from zebrafish embryos, pools of 20 live embryos were homogenized in 800 μl TRIzol Reagent (Invitrogen, Carlsbad, CA, USA) using a homogenizer (IKA T10 basic, IKA-Werke GmbH & Co.KG, Staufen, Germany). The extracted RNA was purified using an RNeasy Mini kit (QIAGEN, Hilden, Germany). The quantity and quality of total RNA were measured using an Agilent RNA 6000 Nano Kit (Agilent). The RIN values for all the RNA samples were higher than 9.0.

Cyanine-3 (Cy3)-labeled cRNA was prepared from 0.1 μg Total RNA using the Low Input Quick Amp Labeling Kit (Agilent), according to the manufacturer's instructions, followed by RNeasy column purification (QIAGEN, Valencia, CA). Dye incorporation and cRNA yield were determined using a NanoDrop ND-2000 Spectrophotometer.

Cy3-labeled cRNA 1.65 μg was fragmented at 60 °C for 30 min in a reaction volume of 25 μl containing 1 × Agilent fragmentation buffer and 2 × Agilent blocking agent following the manufacturer's instructions. Upon completion of the fragmentation reaction, 25 μl of 2 × Agilent hybridization buffer was added to the fragmentation mixture and hybridized to *Danio rerio* (Zebrafish) Oligo Microarray V3 (Design ID: Agilent-026,437) (Agilent) for 17 h at 65 °C in a rotating Agilent hybridization oven. After hybridization, the microarrays were washed for 1 min at room temperature with GE Wash Buffer 1 (Agilent) and for 1 min with 37 °C GE Wash buffer 2 (Agilent).

Slides were scanned immediately after washing on the Agilent SureScan Microarray Scanner (G2600D) using one color scan setting for 4 × 44 array slides (Scan Area 61 × 21.6 mm, Scan resolution 3 μm, Dye channel was set to Green, and Green PMT was set to 100%).

All steps were performed by Takara Bio Inc. (Shiga, Japan), with the exception of the total RNA extraction.

### 3.2 Microarray Data Analysis

The scanned images were analyzed with Feature Extraction Software 12.0.3.1 (Agilent) using default parameters to obtain background-subtracted and spatially detrended processed signal intensities. The processed signal intensities were normalized using the global scaling method. The trimmed mean probe intensity was determined by removing 2% of the lower and the higher ends of the probe intensities to calculate the scaling factor. Normalized signal intensities were then calculated from the target intensity on each array using the scaling factor so that the trimmed mean target intensity of each array was arbitrarily set to 2500.

The signal scores were changed to log2 scores. Differential expression analysis was performed using *limma* package of R [9]. A linear model was fitted to the microarray data and p-values of the empirical Bayes moderated t-statistics test were calculated. Adjusted p-values were calculated using the Benjamini-Hochberg procedure [10] and differentially expressed genes with adjusted p-value ≤ 0.01 were considered significant. The microarray datasets were obtained from three biological replicates. Gene ontology terms [11,12] and Kyoto Encyclopedia of Genes and Genomes (KEGG) pathway IDs (http://www.genome.jp/kegg/) were linked to the microarray information. Volcano plots, PCA plots, and correlation graphs were created in R scripts.

All steps, except for statistical significance tests and figure creation, were performed by Takara Bio Inc. (Shiga, Japan).

## Ethics Statement

The zebrafish experiments were approved by the Hiroshima University Animal Research Committee (Permit Number: F18–2–7).

## CRediT authorship contribution statement

**Masaki Shirai:** Investigation, Data curation, Validation, Formal analysis, Writing – original draft, Writing – review & editing, Visualization. **Nobuyoshi Shimoda:** Investigation, Data curation, Conceptualization, Funding acquisition, Writing – review & editing. **Haruko Takahashi:** Supervision, Investigation, Writing – review & editing. **Kazuya Takayama:** Resources, Investigation. **Yutaka Kikuchi:** Conceptualization, Supervision, Writing – original draft, Writing – review & editing, Funding acquisition, Project administration.

## Declaration of Competing Interest

The authors declare that they have no known competing financial interests or personal relationships that could have appeared to influence the work reported in this paper.

## Data Availability

Expression analysis of WT, dnmt3bb.2-/- and dnmt3aa-/- (Original data) (Gene Expression Omnibus (GEO)).

## Appendix Supplementary materials

Image, application 1

Image, application 2

Image, application 3

Image, application 4

Image, application 5
